# Regional outcome disparities in German head and neck cancer patients: Shorter survival in Eastern Germany

**DOI:** 10.1002/cam4.6690

**Published:** 2023-12-01

**Authors:** Julius M. Vahl, Gabriele Nagel, Tsima Abou Kors, Matthias Brand, Adrian von Witzleben, Michael Sonntag, Ayla Grages, Marie N. Theodoraki, Jens Greve, Michael Denkinger, Dhayana Dallmeier, Christian Idel, Stephan Stilgenbauer, Thomas K. Hoffmann, Simon Laban

**Affiliations:** ^1^ Department of Otorhinolaryngology and Head & Neck Surgery, Head and Neck Cancer Center of the Comprehensive Cancer Center Ulm University Medical Center Ulm Ulm Germany; ^2^ Department of Epidemiology and Medical Biometry University Medical Center Ulm Ulm Germany; ^3^ Agaplesion Bethesda Ulm Institute of Geriatric Research at Ulm University Medical Center and Geriatric Center Ulm Germany; ^4^ Department of Otorhinolaryngology University Hospital Schleswig‐Holstein Lübeck Germany; ^5^ Department of Internal Medicine III Ulm University Medical Center Ulm Germany

**Keywords:** age, cancer registry data, demographic change, socioeconomic health disparity

## Abstract

**Introduction:**

Demographics are important prognostic factors in malignant diseases. A nationwide analysis concerning the prognostic impact of demographics in head and neck cancer (HNC) patients (HNCP) has not been performed previously.

**Methods:**

A retrospective analysis of data from the Center for Cancer Registry Data (ZfKD) and the Federal Statistical Office (Destatis) between 2002 and 2017 was performed. A total of 212′920 HNCP were included. Incidence, tumor stage, age development, sex distribution, age‐, residence‐, and diagnosis‐time‐specific survival were examined.

**Results:**

Mean age of HNCP increased more rapidly than in the general population (slope coefficient: 0.29 vs. 0.20; *p* < 0.0001). Higher age and male sex were associated with a worse prognosis. Whereas overall survival (OS) increased from the early to the later observation period for HNCP <70 years, no OS improvement for HNCP >70 years was found. Furthermore, an OS disadvantage was observed for East Germany compared to West Germany (median 47 vs. 60 months; *p* < 0.0001). This disparity was associated with a disproportionately high ratio of men in East Germany (men/women: 4.4 vs. 3.1; *p* < 0.0001) and a lower mean age (61 vs. 63 years; *p* < 0.0001). In addition to stage, age and sex, residence in East Germany were confirmed as an independent factor for OS in a multivariate analysis.

**Conclusion:**

Finally, three decades after the German reunion, a survival disadvantage for patients in East Germany still exists. This discrepancy may be a result of socioeconomic disparities.

## Graphical abstract

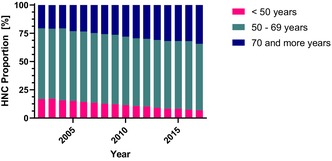

## 1 INTRODUCTION

According to data from the Global Cancer Observatory (GLOBOCAN) in 2020, there were 921′462 reported cases of head and neck cancer (HNC), resulting in 447′307 deaths worldwide.^1^ The disease exhibited a male‐to‐female ratio of 3:1.^1^ In addition to well‐known risk factors such as smoking, alcohol consumption, and human papillomavirus (HPV) infections,^2^, ^3^, ^4^ age plays a significant role in increasing the susceptibility of various tissues to cancer development due to cellular damage accumulation and alterations in the endocrine and immune systems.^5^

For the purposes of our study, we focused specifically on Germany, a central European country bordered by nine nations. The country's geography varies, with the northern part characterized by low‐lying plains and coastal areas along the Baltic and North Seas, while the southern regions are dominated by the Bavarian hills and Alps. Prior to the reunification in 1990, Germany was divided into two entities: the Federal Republic of Germany (BRD) in the West and the German Democratic Republic (DDR) in the East. Consequently, different political, economic, and social systems evolved in the East and West over the years.

Presently, high German smoking rates (27% of men and 19% of women smoked daily in 2019^6^) and an increasing prevalence of HPV (up to 50%) in oropharyngeal cancers have been observed in East Germany.^7^ The percentage of elderly and multimorbid patients is rising, resulting in higher healthcare costs throughout Germany.^8^ Additionally, older patients face a greater risk of cancer‐related mortality and may not receive the full standard treatment due to various factors. As a result, they may not benefit from advancements in treatment that have occurred over the past two decades.^9^ Recently, our research revealed a significant increase in the annual incidence rates and mean age of patients at a tertiary cancer center in Germany, particularly among those aged over 70 years.^10^ Gender has been recognized as a prognostic factor,^11^ and the region of residence may be associated with socioeconomic status, which is also considered an important prognostic factor.^12^ However, despite available data from specific regions of Germany or previous evaluations of cancer registry data, nationwide analyses exploring the prognostic association of demographic factors in Germany are lacking.^13^, ^14^, ^15^

Thus, the objective of our study was to determine the impact of age, gender, and region of residence on the overall survival (OS) of HNC patients in a comprehensive German nationwide dataset.

## 2 PATIENTS AND METHODS

### 2.1 Data collection

We requested anonymized data from the German Center of Cancer Registry (‘Zentrum für Krebsregisterdaten’ (ZFKD)) of patients newly diagnosed with HNC between 2002 and 2017.^16^ We received data of 212′920 HNCP including sex, anonymized date of birth, county affiliation, rounded date of diagnosis, TNM (tumor, nodal, metastasis) status, and date of mortality follow‐up (East Germany: December 2015, West Germany: December 2017) with the endpoint vital status. Information on Epstein–Barr virus or human papillomavirus was not available. Furthermore, we requested data concerning age and sex of the general German population in between 2002 and 2017 at the central information service of the State Statistical Office Baden‐Wuertemberg (‘Statistisches Bundesamt, Zentraler Auskunftsdienst’), which afterwards coordinated the data retrieval from the 16 individual countries and their statistical offices.

### 2.2 Data exclusion

For the analysis of HNC incidence, TNM status and demographics of HNCP we were able to use the major part of the data set (*n* = 212′920). Nevertheless, sometimes data of TNM status was missing (Table 1, Table S1). For 45.1% of HNCP no UICC stage could be created because of at least partly missing TNM status (mostly M status). The UICC stage calculation was based on the 8th version, which was simplified to four stages without subgroups and in which all oropharyngeal carcinomas were included according to p16‐negative classification (because of missing information of HPV status in registry data). It is also of importance that the federal state of Baden‐Wuerttemberg started reporting data to the cancer registry beginning in 2009 after reporting of cancer registry data became obligatory, legal requirement (Federal Cancer Registry Data Law; 18.08.2009). A delay in reporting seemed likely to occur in 2016 and 2017. In fact, no HNC reports were received from East Germany in 2017. Therefore, we have not analyzed TNM status, mean age and sex of HNCP development in relation to residence during the years 2016 and 2017.

**TABLE 1:** Cohort characteristics. An overview of the entirety of all included HNCP in terms of their primary sites, stage, gender, mean age, age group and residence is provided.

	Total number and percent		Gender		Mean age and standard deviation		Age group			Residence	
			Men	Women			<50 years	50–69 years	≥70 years	East	West
Primary site											
Oropharynx	66,276	31.1	49,985	16,291	61.9	10.6	7938	43,627	14,711	16,910	49,366
Oral cavity	30,011	14.1	21,135	8876	62.9	11.8	3947	17,961	8103	7473	22,538
Larnyx	52,480	24.6	45,527	6953	65.6	10.8	3817	30,264	18,399	11,431	41,049
Hypopharynx	25,080	11.8	21,574	3506	62.3	10.0	2676	16,697	5707	6181	18,899
Nasopharynx	5240	2.5	3781	1459	58.5	15.3	1346	2677	1217	1099	4141
Others	33,833	15.9	22,132	11,701	63.4	12.9	4759	18,625	10,449	7139	26,694
Stage											
UICC I	22,041	10.4	16,787	5254	63.0	11.3	2720	13,054	6267	6936	15,105
UICC II	14,199	6.7	10,784	3415	62.6	11.2	1762	8744	3693	4566	9633
UICC III	19,164	9.0	14,646	4518	61.9	11.1	2588	12,053	4523	5938	13,226
UICC IV	61,536	28.9	49,600	11,936	61.4	10.3	7974	40,845	12,717	20,289	41,247
Missing (no full TNM)	95,980	45.1									

*Note*: ‘Others’ include for example, salivary glands or sinunasal cancers.

For survival analyses HNCP with missing data on survival time and status were excluded. In addition, patients marked as deceased, but without a date of death and those alive, but without a date of last follow‐up or DCO (death certificate only) cases were excluded for survival analysis too. Hence, the cohort available was reduced to *n* = 193′877.

### 2.3 Data analysis

Inhomogeneous data sets from the statistical offices were harmonized and combined in Microsoft Excel 2019. The German Center for Cancer Registry delivered summarized data in an excel file. Data were imported to IMB SPSS Statistics 26 and GraphPad Prism 9 for statistical testing. Figures were arranged in GraphPad Prism 9 and Microsoft Power Point 2019 and the tables were created in Microsoft Excel 2019.

### 2.4 Statistical analysis

To check whether mean values among two groups were significantly different, we first tested normality by Shapiro–Wilk test (*n* ≤ 50) or Kolmogorov–Smirnov test (*n* > 50) and homoscedasticity by Levene's test. Unpaired *t*‐test was performed if normal distribution and homoscedasticity were present or alternatively a Welch test if normal distribution was given but homoscedasticity was absent. In cases of missing normality, we applied nonparametric Mann–Whitney *U* test to compare scaled values among two groups. Differences of slopes, assuming a linear relationship, were tested with Deming regression analysis. Deviation of the slope coefficient from zero was then tested by *F* test. Standard deviation was abbreviated with ‘SD’. Chi‐squared test was used to examine differences in distribution of categorial variables among large sample sizes. Survival data was generated by Kaplan–Meier estimator. Differences in survival data were tested with (pairwise) Log rank tests. Standard error was abbreviated with ‘SEM’. 95% confidence intervals (CIs) were given. Multivariate analysis was performed by Cox regression with backward selection of variables after model fitting by Omnibus tests providing hazard ratios (HR) with 95% CI and level of significance (*p*).

## 3 RESULTS

### 3.1 Patients' characteristics

A total of 212′920 cases were included in the analysis of German cancer registry data between the years 2002 and 2017 (Table 1). The mean age in the cohort was 63.2 years (SD = 11.4). The ratio of men to women was 3.4. Most patients had oropharyngeal (31.1%) and laryngeal cancers (24.6%), locally limited (T1, T2) primary tumor status (41.9%), presence of regional lymph node metastases (38.3%) and no distant metastases (54.8%) at initial diagnosis (Table S1). However, incomplete data were frequent (T: 23.1%; *N*: 32.8%; *M*: 41.8%). The largest group of HNCP (28.9%) had UICC Stage IV at initial diagnosis (missing data in 45.1%). Median survival in the total cohort was 57 months (SEM = 0.4; CI: 56.3–57.7).

### 3.2 Increasing proportion of older HNCP

**FIGURE 1:**
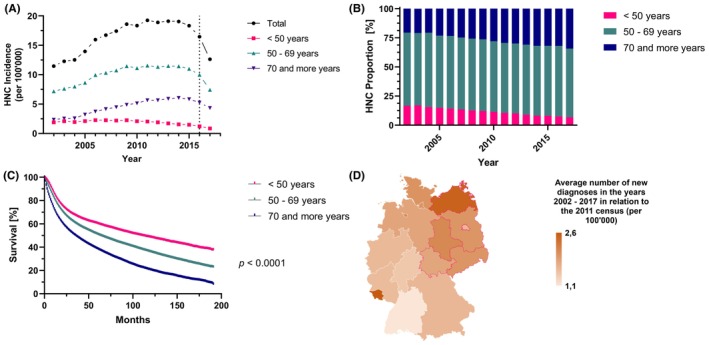
Head and neck cancer incidence and age‐specific survival in Germany. (A) Newly diagnosed cases of HNC per 100,000 inhabitants in Germany between the years 2002 and 2017 are displayed with a subdivision into three age‐related groups. Incomplete data transmission to the cancer registry is assumed since 2016 (indicated with a dotted, vertical line). (B) The relative, age‐group specific proportion of HNC incidence during the observation period is shown. (C) Age‐group specific overall survival is presented. (D) The mean number of newly diagnosed HNC per federal state is demonstrated in relation with the resident population numbers from the census in 2011.

The incidence per 100′000 inhabitants was calculated in relation to data from the State Statistical Office for the general population in Germany for each year between 2002 and 2017. The incidence of HNC was rising from 11.5 in 2002 to 18.3 in 2015 and followed by a decrease to 12.6 in 2017 (Figure 1A). Due to late initiation of data reporting from Baden Württemberg to the cancer registry and a reporting delay renders incidence rates after 2015 unreliable. A clear and steady increase in the proportion of older HNCP (≥ 70 years) between 2002 and 2017 from 20.6% to 34.4% (Figure 1B) was observed. In the same time period, the proportion of younger HNCP (< 50 years) decreased from 16.7% to 6.8%, whereas the proportion of HNCP 50–69 years old remained stably between 62.7% and 58.9%. Meanwhile, OS was significantly shorter in the two older age groups compared to younger patients (Figure 1C). The median survival is 109 months (SEM = 2.1; CI: 104.8–113.2) for young (<50 years), 63 months (SEM = 0.5; CI: 62.0–64.0) for medium old (50–69 years) and 35 months (SEM = 0.4; CI: 34.2–35.8) for old (≥70 years) HNCP (*p* < 0.0001). The mean incidence in relation to the population numbers of the 2011 census did not differ significantly (*p* = 0.33) between the western and eastern federal states (Figure 1D). However, certain regional differences in the incidence rate can be noted.

### 3.3 Outcomes in patients diagnosed after 2009 improved in younger patients only

**FIGURE 2:**
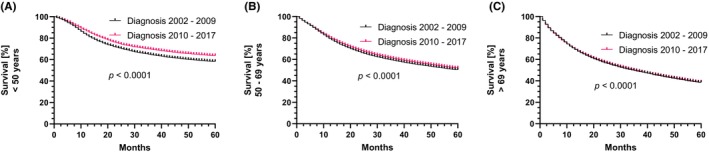
Comparison of time‐specific survival. Age‐specific survival over 5 years is displayed for patients diagnosed between 2002 and 2009 in comparison to those diagnosed between 2010 and 2017 for (A) patients <50 years of age at diagnosis, (B) patients > = 50 years, but <70 years of age at diagnosis and (C) patients >70 years of age at diagnosis.

To assess the impact of changes in standard treatment during the first half of the observation period, the cohort was divided by the year of initial diagnosis (period 1: 2002–2009 vs. period 2: 2010–2017). The mean OS of all HNCP diagnosed between 2002 and 2009 considering a follow‐up period of 5 years was 39.3 months (SEM = 0.1; CI: 39.3–39.6)) compared to 39.5 months (SEM = 0.1; CI: 39.4–39.7) in patients diagnosed between 2010 and 2017. However, when analyzed separately for the three age groups within these two intervals a significant improvement in mean survival was observed for young HNCP (< 50 years; period 1: 43.1 months vs. period 2: 45.5 months *p* <; 0.0001; Figure 2A) and for middle aged HNCP (50–69 years; period 1: 40.2 months vs. period 2: 41.0 months; *p* < 0.0001; Figure 2B). But no significant difference was detected between the two time periods for old HNCP (≥70 years; period 1: 34.6 months vs. period 2: 34.6 months; *p* = 0.76; Figure 2C).

### 3.4 TNM and stage at diagnosis was constant over time

During the whole observation period, the ratio of advanced to early primary tumor status (T3 + 4 / T1 + 2), N+ to N0 and M1 to M0 of newly diagnosed HNC did not change significantly (Figure S1A). However, HNCP in East Germany consistently presented with higher T status (*p* < 0.0001) and N status (*p* < 0.0001), but not M status (*p* = 0.15). Differences in UICC stage between East and West (Figure S1B) were not observed (*p* = 0.22). Moreover, the portions of different HNSCC entities differed only marginally, but still significantly (p < 0.0001) by a maximum of 3% in East and West Germany. As expected, UICC stage was strongly associated with prognosis (Figure S1c). Median survival was 127 months (SEM = 1.6; CI: 123.8–130.2) in Stage I, 94 months (SEM = 1.5; CI: 91.0–97.0) in Stage II, 71 months (SEM = 1.2; CI: 68.6–73.4) in Stage III and 29 months (SEM = 0.3; CI: 28.4–29.6) in Stage IV HNCP (*p* < 0.0001).

### 3.5 Mean age of HNCP was rising, especially in West Germany

**FIGURE 3:**
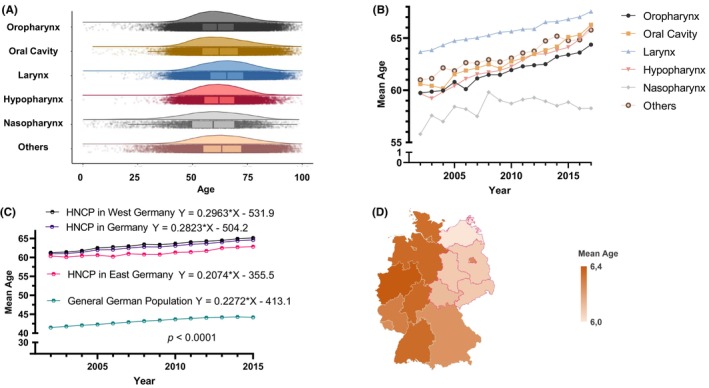
Mean age development by tumor entity and residence. (A) The age distribution of HNCP is displayed as box and violin plots by primary tumor site. (B) Mean age during the observation period is shown by primary site. (C) Mean age of all HNCP per year during the observation period compared to those diagnosed in East or West Germany and age development among the general population. The significance level (*p*) for the comparisons between slope coefficients is displayed as well. (D) A geographical illustration of the regional mean age of HNCP in Germany for the 16 federal states is shown.

The mean age of all included HNCP (Figure 3A,D) during the observation period was 63.2 years (SD = 11.4). In the meantime, the age of the general population was between 41.5 and 44.4 years (Figure 3C). The highest mean age was observed in laryngeal cancer patients (65.6 years; SD = 10.8) and the lowest mean age in nasopharyngeal cancer patients (58.5 years; SD = 15.3). Mean age increased significantly over time in all HNC primary tumor sites, whereas the lowest increase was observed in nasopharyngeal cancer patients (Figure 3B). In comparison to the age increment in the general population, mean age among all HNCP was increasing at a significantly higher rate (slope coefficient 0.3 vs. 0.2; *p* < 0.0001; Figure 3C). The increase in mean age among HNCP was significantly faster in the West than in the East of Germany (slope coefficient: 0.3 vs. 0.2; *p* < 0.0001; Figure 3C). Consistent with this observation, mean age among HNCP in the East was 2.5 years lower than in the West (61.3 years (SD = 11.2) vs. 63.8 (SD = 11.4); *p* < 0.0001; Figure 3D).

### 3.6 Male sex and residence in East Germany were associated with poor survival

**FIGURE 4:**
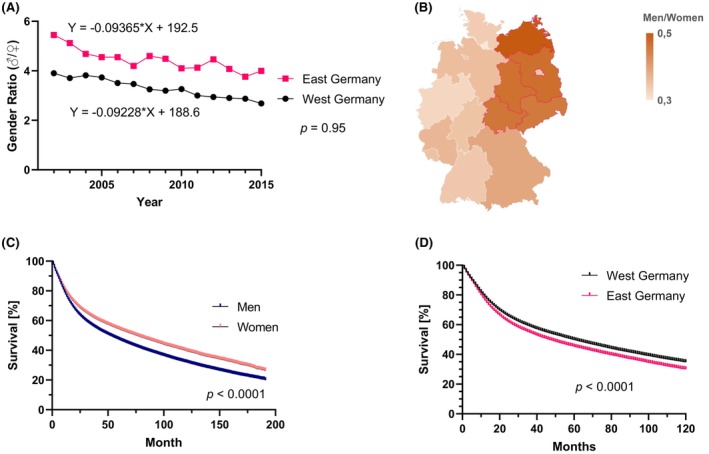
Gender distribution and association with imbalance in survival in Eastern and Western Germany. (A) The gender ratio (men/women) in East and West Germany is displayed over time between 2002 and 2015. The significance level (p) for the comparison between those two slope coefficients is displayed. (B) The mean gender ratio is illustrated geographically for each of the 16 federal states. (C) The overall survival in relation to patient sex is shown with *p*‐value resulting from log rank test. (D) The survival over 10 years by region of residence is shown with the *p*‐value from log rank testing.

The sex ratio (men/women) among HNCP was significantly higher in East than in West Germany (mean ratio: 4.4 vs. 3.3; *p* < 0.0001; Figure 4A,B). However, the sex ratio has decreased in both parts of Germany evenly (*p* = 0.95) over time (Figure 4A). Male sex was significantly associated with shorter OS (male: 52 months vs. female: 76 months; *p* < 0.0001; Figure 4C). Residence in East Germany was also associated with significantly shorter median OS (East Germany: 47 months vs. West Germany: 60 months; *p* < 0.0001; Figure 4D).

### 3.7 Multivariate analysis confirmed impact of demographic factors on prognosis

**FIGURE 5:**
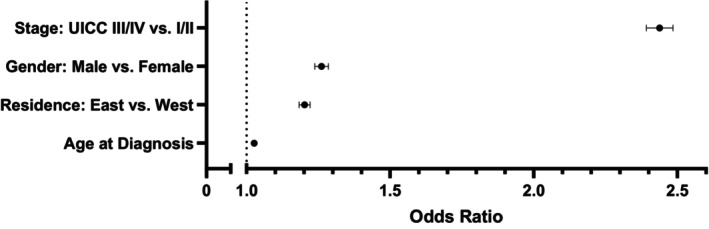
Multivariate Cox regression of stage and demographic factors. Hazard ratios with 95% CI (whiskers) for tumor stage, gender, place of residence and age at diagnosis are shown from a multivariate cox regression analysis. A hazard ratio of 1 is indicated by the dotted, vertical line.

Finally, we performed a multivariate cox regression analysis considering UICC stage, age, sex and residence (East vs. West). All factors in the model were confirmed as independent factors for prognosis (Figure 5). The highest HR was found for advanced UICC Stages III, IV (HR: 2.43; 95% CI: 2.39, 2.49; *p* < 0.0001), followed by male sex (HR: 1.26; 95% CI: 1.24, 1.29; *p* < 0.0001), residence in East Germany (HR: 1.20; 95% CI: 1.18, 1.22; *p* < 0.0001) and higher age at diagnosis (HR: 1.03; 95% CI: 1.03, 1.03); *p* < 0.001). It is worth emphasizing that survival was inferior in the East Germany for each of the tumor entities studied here (Table S2).

## 4 DISCUSSION

This is the first comprehensive, nation‐wide analysis of demographic factors on the prognosis in head and neck cancer. Our study confirms reports about increasing age in HNCP from the United States and parts of Germany.^10^, ^17^ This poses a challenge for health care systems due to a higher degree of morbidity and mortality associated with treatment^9^, ^18^, ^19^, ^20^ and is associated with a significant financial burden on health care systems.^8^ Frailty is a predictor of severity for complications in HNCP undergoing surgery^21^ and surgery is less often performed in older patients.^9^ A greater increase in mean age in comparison to the demographic development in the general population underlines the need for age‐specific assessments to improve cancer care^22^, ^23^ and addresses the described outcome disparities by age. At the same time, the decreasing proportion of younger HNCP may be a result of primary cancer prevention efforts such as anti‐smoking and anti‐alcohol abuse campaigns.^24^ Unfortunately, there is no HNC screening tool yet, which is also reflected in the constant tumor burden at initial diagnosis during our observation period.

There were significant improvements in the clinical management of HNC during the first half of the observation period (2002–2009) with regard to definitive chemoradiation,^25^, ^26^, ^27^, ^28^ adjuvant (chemo‐)radiation^29^, ^30^, ^31^ and palliative chemotherapy.^32^ These innovations may explain the increased OS in younger and middle‐aged patients. However, most of these innovations consisted of treatment intensification, from which older patients seem not to have had a similar benefit.^33^, ^34^ This is also in line with earlier (1996–2005) evaluations of the Thuringian cancer registry.^15^ In older patients, molecular biology, treatment goals and available options may differ significantly from younger patients.^9^, ^35^, ^36^ Thus, there is a definitive need for clinical trials focusing on treatment of older patients.

An additional prognostic factor was sex, increasingly emphasized by the emerging field of sex medicine, also in oncology.^11^, ^37^ The ratio of male to female patients has been decreasing over the observation period. Potentially due to higher degrees of smoking and alcohol abuse in male patients, these seem to have a higher risk of death. Primary prevention strategies include prophylactic HPV vaccination which will in the future contribute to a decreasing incidence in both male and female patients, especially if vaccination rates can be improved further (approval in Germany for boys since 2018 and for girls already since 2007^38^).

An alarming result of our analysis is the outcome disparity between East and West Germany. More than 30 years after the German reunion (joining of the DDR to the BRD: 03.10.1990), such a difference in OS among patients indicates the need for health policy action. Both, a more advanced tumor stage at diagnosis and a higher fraction of male patients seem to contribute to this survival difference, whereas the younger mean age of patients in East Germany could counterbalance these influences. Nevertheless, residence in Eastern Germany was confirmed as an additional, independent factor by multivariate analysis.

Thereupon, persisting gaps in general life expectancy between East and West have been reported, but seem to be slowly vanishing after reunification.^39^, ^40^ At the beginning of our study in 2002, the difference in life expectancy in the general population amounted to 0.4 years for women and 1.5 years for men.^39^ A big part of the improved life expectancy in the East since then is considered to be a result of a decline in cardiovascular mortality.^39^, ^40^ However, socioeconomic factors also exist that may have a strong impact on HNCP and other cancer patients' mortality. In fact, socioeconomic disadvantages such as differences in education, occupational status and income can lead to differences in experienced health burden (e.g. work related) and resilience. These effects may be amplified by differences in access to health care and personal behavior (nutrition, substance abuse, compliance etc.).^41^, ^42^ In addition, the healthcare system in the East is particularly dependent on modernization.^43^ Inequalities in health may then even further increase socioeconomic imbalance.^42^ In Germany, the mean household income among inhabitants in East Germany is lower and there is a higher rate of unemployment^39^ indicating an elevated level of socioeconomic stress which may result in a higher risk for substance abuse.^44^ Similar difficulties seem to be experienced in the USA, here, data on reduced survival of black patients are available; the reasons are very diverse as well.^45^ Further, the migration of well‐educated inhabitants to other parts of Germany, a shrinking population with a higher proportion of older inhabitants and an underdeveloped public transport infrastructure, especially in the rural areas may further destabilize comprehensive health care.^39^, ^46^, ^47^, ^48^ Current efforts to equalize these disparities between East and West Germany need to be continued and expanded. In this manner, the consolidation of a welfare state, facilized health care access, and an enhanced political inclusion of citizens are thought to be helpful.^49^ In addition, a subsequent extension of this study to urban and rural regions in Germany could provide further insights.^50^

Finally, this study shows the need to enhance cancer data availability and accuracy of documentation. Despite of the mandatory reporting of cancer‐related data to cancer registries in Germany since 2009, collected data still have a high prevalence of missing data.^51^ However, the relations between T, N, M status collected here are comparable to U.S. cancer registry evaluations.^45^ Efforts to specialize care within dedicated head and neck cancer centers may in the future improve data completeness. However, this constrained data recording is similar to other countries.^52^ In addition, available data from the statistical offices of the federal were not structured uniformly and represent a significant hurdle for data analysis in public health research.

## 5 CONCLUSIONS

Age, sex, and patient residence contribute independently to the prognosis of HNCP. Outcome disparities between East and West Germany need to be put on the agenda by health policy makers. Supporting well‐networked head and neck cancer centers to improve access to specialized care, the use of geriatric assessment tools and addressing socioeconomic imbalances may be key to improve treatment outcomes.

## AUTHOR CONTRIBUTIONS

**Julius M. Vahl:** Conceptualization (equal); data curation (lead); formal analysis (equal); investigation (equal); methodology (equal); visualization (equal); writing – original draft (equal). **Gabriele Nagel:** Formal analysis (equal); investigation (equal); methodology (equal); writing – review and editing (equal). **Tsima Abou Kors:** Investigation (supporting); visualization (equal); writing – review and editing (equal). **Matthias Brand:** Writing – review and editing (supporting). **Adrian von Witzleben:** Writing – review and editing (equal). **Michael Sonntag:** Writing – review and editing (equal). **Ayla Grages:** Writing – review and editing (equal). **Marie N. Theodoraki:** Writing – review and editing (equal). **Jens Greve:** Writing – review and editing (equal). **Michael Denkinger:** Conceptualization (supporting); investigation (supporting); methodology (supporting); writing – review and editing (supporting). **Dhayana Dallmeier:** Conceptualization (supporting); investigation (supporting); methodology (supporting); writing – review and editing (supporting). **Christian Idel:** Conceptualization (supporting); investigation (supporting); writing – review and editing (supporting). **Stephan Stilgenbauer:** Writing – review and editing (equal). **Thomas K. Hoffmann:** Resources (lead); supervision (supporting); writing – review and editing (equal). **Simon Laban:** Conceptualization (lead); data curation (supporting); formal analysis (equal); investigation (equal); methodology (lead); project administration (lead); resources (equal); supervision (lead); validation (lead); visualization (equal); writing – original draft (equal); writing – review and editing (equal).

## FUNDING INFORMATION

No funding was received.

## CONFLICT OF INTEREST STATEMENT

Simon Laban: Advisory Boards: Merck Sharp & Dohme (M.S.D.), Bristol Myers, Squibb (B.M.S.), Astra Zeneca (A.Z.). Honoraria: M.S.D., B.M.S., A.Z., Merck Serono. Thomas K. Hoffmann: Advisory Boards: M.S.D., B.M.S., Sanofi. Honoraria: M.S.D., B.M.S., Merck Serono. All other authors declared no conflict of interests in conjunction with this work.

## ETHICS STATEMENT

All procedures performed were in accordance with the ethical standards of the institutional research committee and with the 1964 Helsinki declaration and its later amendments or comparable ethical standards. In agreement with our ethics committee of the University Ulm, Germany, no designated ethic vote was needed because data was anonymized before analysis.

## INFORMED CONSENT

Epidemiological cancer registration in Germany is regulated by state laws and data received were anonymized facilized. The Federal Cancer Registry Data Act of 2009 defines the tasks of the Center for Cancer Registry Data at the Robert Koch Institute as the national evaluation center. This means that all physicians and dentists in the state are required to report cancer cases they are involved in diagnosing, treating or following up to the state cancer registry. Patients consent is not required for this.

## Supporting information


Figure S1.

Table S1.

Table S2.

## Data Availability

The original dataset cannot be shared. The data on HNCP in Germany have been requested in a multistep process from the German cancer registry for a dedicated purpose and specific group of persons only and must not be shared. The data on the general population was ordered from de federal statistical office and suboffices at a charge and may only be used applicant‐related too.
