# Supplementary figures and images for: Regional outcome disparities in German head and neck cancer patients: Shorter survival in Eastern Germany
Source: Cancer Med. 2023 Dec 1;12(23):21426–35. doi: 10.1002/cam4.6690 (PMC10726835; doi:10.1002/cam4.6690)

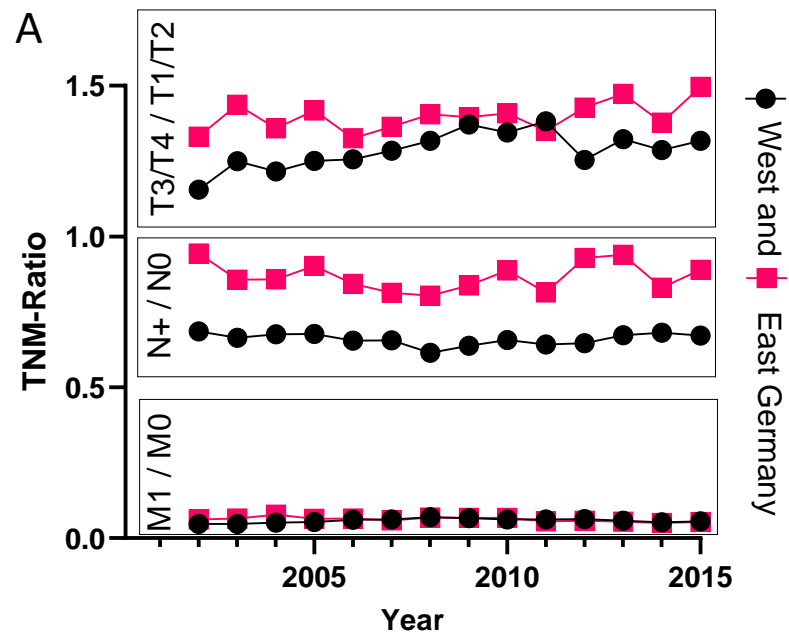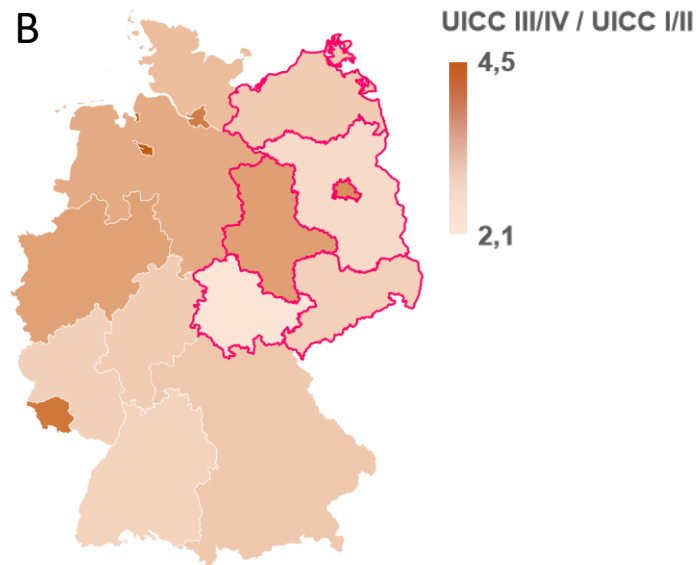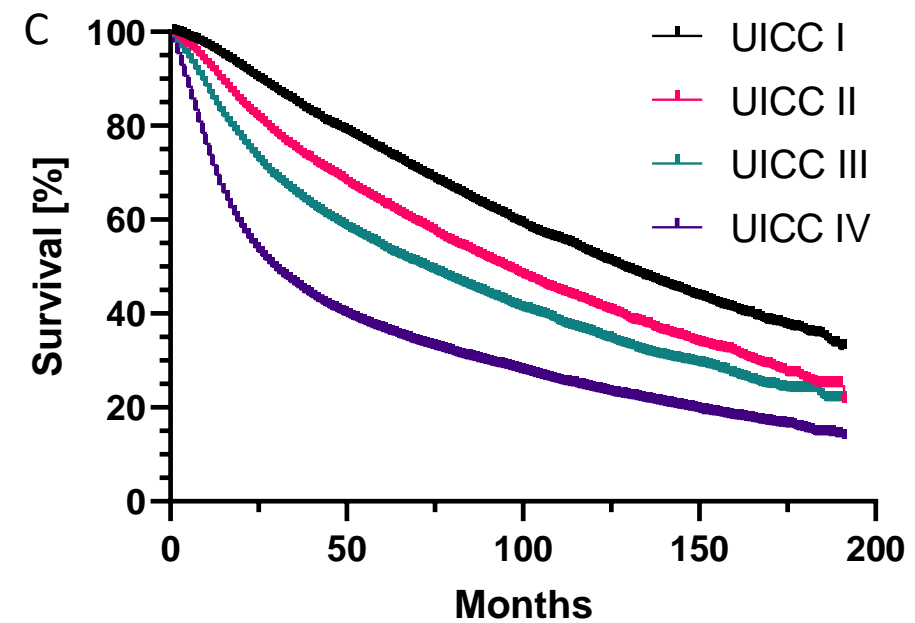

Supplement: Supplementary file 1 — Figure S1. [file CAM4-12-21426-s002.pdf]
