# Supplementary material for: Regional outcome disparities in German head and neck cancer patients: Shorter survival in Eastern Germany
Source: Cancer Med. 2023 Dec 1;12(23):21426–35. doi: 10.1002/cam4.6690 (PMC10726835; doi:10.1002/cam4.6690)
Supplement: Supplementary file 3 — Table S2. [file CAM4-12-21426-s001.docx]

| *Primary Site* | *Median Overall Survival [Month]*  *with SEM and CI* | | *p-Value* |
| --- | --- | --- | --- |
|  | *East* | *West* |  |
| Oropharynx | 46 (1.1; 43.9 – 48.1) | 58 (0.8; 56.4 – 59.6) | p < 0.00001 |
| Oral Cavity | 47 (1.6; 43.8 – 50.2) | 59 (1.2; 56.7 – 61.3) | p < 0.00001 |
| Larynx | 72 (1.6; 69.0 – 75.1) | 85 (1.1; 82.9 – 87.1) | p < 0.00001 |
| Hypopharynx | 21 (0.5; 20.0 – 22.0) | 25 (0.4; 24.2 – 25.8) | p < 0.00001 |
| Nasopharynx | 61 (7.4; 46.5 – 75.5) | 89 (4.4; 80.3 – 97.7) | p = 0.0007 |
| Others | 50 (1.9; 46.3 – 53.7) | 63 (1.2; 60.6 – 65.4) | p < 0.00001 |
