# Supplementary material for: Regional outcome disparities in German head and neck cancer patients: Shorter survival in Eastern Germany
Source: Cancer Med. 2023 Dec 1;12(23):21426–35. doi: 10.1002/cam4.6690 (PMC10726835; doi:10.1002/cam4.6690)
Supplement: Supplementary file 3 [file CAM4-12-21426-s003.docx]

		Total Number and Percent	
**T**	T1	46891	22.0
	T2	42232	19.8
	T3	28569	13.4
	T4	35990	16.9
	Tx	10132	4.8
	missing Data	49106	23.1
**N**	N0	61362	28.8
	N1	19756	9.3
	N2	56212	26.4
	N3	5658	2.7
	missing Data	69932	32.8
**M**	M0	116743	54.8
	M1	7199	3.4
	missing [illegible]	88978	41.8
